# Loss of IRF7 accelerates acute myeloid leukemia progression and induces VCAM1-VLA-4 mediated intracerebral invasion

**DOI:** 10.1038/s41388-022-02233-w

**Published:** 2022-03-07

**Authors:** Hao Wang, Dongyue Zhang, Xiaoxi Cui, Yibo Dai, Chenchen Wang, Wenli Feng, Xiaoqian Lv, Yifei Li, Lina Wang, Yongxin Ru, Yingchi Zhang, Qian Ren, Guoguang Zheng

**Affiliations:** State Key Laboratory of Experimental Hematology, National Clinical Research Center for Blood Diseases, Haihe Laboratory of Cell Ecosystem, Institute of Hematology & Blood Diseases Hospital, Chinese Academy of Medical Sciences & Peking Union Medical College, 288 Nanjing Road, Tianjin, 300020 China

**Keywords:** Cancer models, Cancer stem cells, CNS cancer, Acute myeloid leukaemia

## Abstract

Interferon regulatory factor 7 (IRF7) is widely studied in inflammatory models. Its effects on malignant progression have been documented mainly from the perspective of the microenvironment. However, its role in leukemia has not been established. Here we used MLL-AF9-induced acute myeloid leukemia (AML) mouse models with IRF7 knockout or overexpression and xenograft mouse models to explore the intrinsic effects of IRF7 in AML. AML-IRF7^−/−^ mice exhibited accelerated disease progression with intracerebral invasion of AML cells. AML-IRF7^−/−^ cells showed increased proliferation and elevated leukemia stem cell (LSC) levels. Overexpression of IRF7 in AML cells decreased cell proliferation and LSC levels. Furthermore, overexpression of transforming growth-interacting factor 1 (TGIF1) rescued the enhanced proliferation and high LSC levels caused by IRF7 deficiency. Moreover, upregulation of vascular cell adhesion molecule 1 (VCAM1), which correlated with high LSC levels, was detected in AML-IRF7^−/−^ cells. In addition, blocking VCAM1-very late antigen 4 (VLA-4) axis delayed disease progression and attenuated intracerebral invasion of AML cells. Therefore, our findings uncover the intrinsic effects of IRF7 in AML and provide a potential strategy to control central nervous system myeloid leukemia.

## Introduction

Acute myeloid leukemia (AML) is characterized by the accumulation of a large number of immature myeloid cells [1] and differs from lymphoblastic leukemia in clinical feature and treatment [2, 3]. Both intrinsic and extrinsic abnormalities participate in the progression of AML [4, 5] by affecting the characteristics of the malignant population, including leukemia stem cell (LSC) levels [6], proliferation [7], dissemination, and infiltration [1], etc. Specifically, infiltration into the central nervous system (CNS) not only enables AML cells to escape the effects of chemotherapy due to the blood-brain barrier (BBB) [8] but also increases the risk of neurocognitive deficits and secondary malignancies after treatment [9]. Elucidating the mechanisms of disease progression will provide a basis for the diagnosis and treatment of AML.

The interferon regulatory factor (IRF) family contains 9 members (IRF1 to IRF9), which play pivotal roles in both innate and adaptive immunity [10]. They also act as intrinsic or microenvironmental factors in malignant progression [10, 11]. IRF7 has been widely studied in inflammatory models [12], while its suppressive effects in malignancies are mainly accomplished by microenvironmental immune cells, including granulocytic myeloid derived suppressor cells [13], macrophages [14], T cells, and natural killer (NK) cells [15, 16]. Its intrinsic effects in tumor progression seem to be cell-type dependent as opposite effects were reported in breast cancer [17] and glioblastoma [18]. However, the mechanism has not been established. Importantly, the intrinsic effects of IRF7 in AML remain unknown.

Transforming growth-interacting factor 1 (TGIF1) belongs to the three-amino acid loop extension family of homeodomain proteins and is primarily described as a transcriptional repressor in the transforming growth factor-β (TGF-β) signaling pathway [19]. TGIF1 plays important roles in normal and malignant hematopoiesis. TGIF1^−/−^ hematopoietic stem cells (HSCs) are less proliferative, more quiescent, and have higher repopulation potential [20]. The expression of TGIF1 was decreased in MLL-rearranged AML patients [21] and overexpression of TGIF1 promoted cell differentiation and cell cycle exit in vitro and prolonged the survival in vivo [21, 22]. It was also reported that knockdown of TGIF1 resulted in the inhibition of proliferation and differentiation in myeloid leukemia-derived cell lines [23]. The regulatory network of TGIF1 is not fully understood, and the link between IRF7 and TGIF1 has not been established.

Malignant cells capable of crossing the BBB and forming brain metastases acquire a more malignant phenotype [9]. In leukemia, studies have focused on lymphocytic leukemia, whereas studies using AML models are scant. Cytokines, chemokines, and adhesion molecules participate in this multiple-step pathologic process [24–26]. Specifically, the interaction between the very late antigen 4 (VLA-4, integrin α4β1) in leukemia cells and vascular cell adhesion molecule 1 (VCAM-1) in endothelial cells is essential for lymphocytic leukemia cells to cross the BBB [27]. This axis not only mediates the adhesion step but also initiates essential signaling within endothelial cells and promotes the identification of optimal sites for transmigration [28]. However, whether this axis facilitates the development of myeloid CNS leukemia (CNSL) is still unclear.

In this study, MLL-AF9-induced mouse AML models with IRF7 knockout or overexpression and xenograft mouse models were used to explore the intrinsic effects of IRF7 in AML. The deficiency of IRF7 in AML cells accelerates disease progression by promoting cell proliferation and elevating LSC levels. These adverse phenotypes are mediated by the downregulation of TGIF1. Furthermore, knockout of IRF7 causes intracerebral invasion of AML cells. Blocking VCAM1-VLA-4 axis delayed disease progression and attenuated intracerebral invasion of AML cells.

## Results

### AML-IRF7^−/−^ mice exhibit accelerated disease progression

**Fig. 1:**
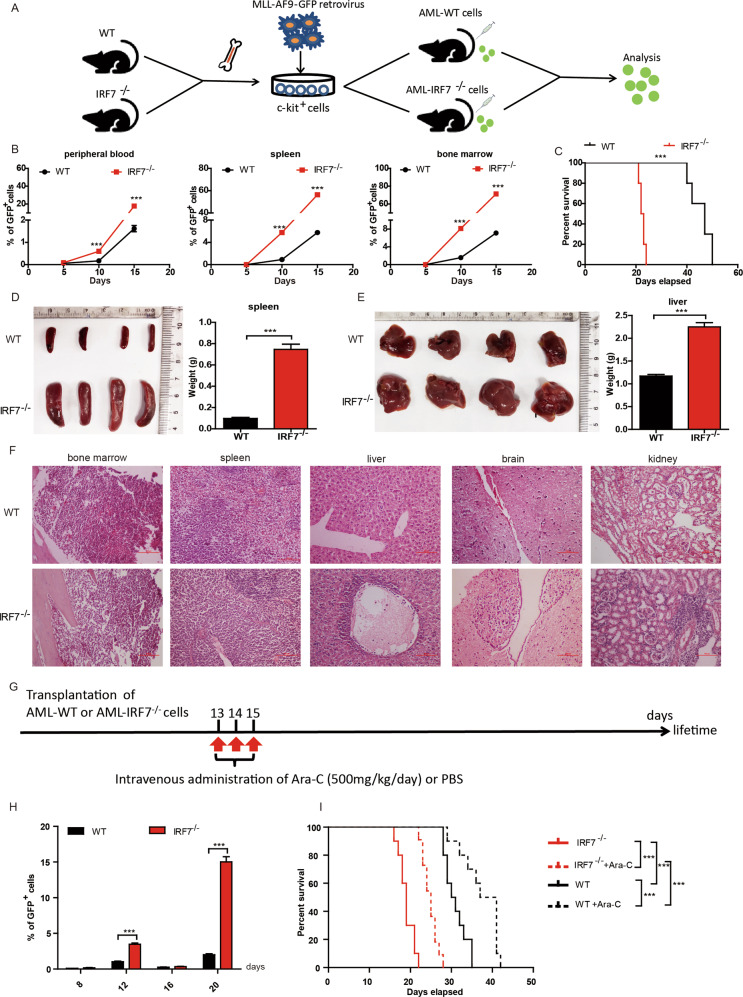
AML-IRF7^−/−^ mice exhibit accelerated disease progression. **A** Schematic overview of the development of the MLL-AF9-induced AML-WT and AML-IRF7^−/−^ mouse models. **B**–**F** The disease progression of the two models was studied when 1 × 10^6^ AML cells were transplanted into recipient mice. **B** The percentage of GFP^+^ cells in peripheral blood, spleen, and bone marrow was monitored every 5 days (*n* = 4). **C** The survival of the mice is shown in Kaplan–Meier curves (*n* = 10 for each group). **D**, **E** The mice were sacrificed on day 15. The size and weight of the spleens (**D**) and livers (**E**) are shown, and HE-stained sections of bone marrow, spleen, liver, brain, and kidney were observed under a light microscope. **F** Scale bars, 100 μm. **G**–**I** The mice were transplanted with 1 × 10^6^ AML cells followed by Ara-C treatments at the indicated time points. **G** Schematic overview of the experiments. **H** The percentage of GFP^+^ cells in peripheral blood was monitored on days 8, 12, 16, and 20. **I** The survival of the mice is shown in Kaplan–Meier curves (*n* = 10 for each group). Data are presented as mean ± S.E.M. Unpaired Student’s *t* test, one-way ANOVA tests and Kaplan–Meier estimates were used. ***p < 0.001.

MLL-AF9-induced AML models were established by using WT and IRF7^−/−^ mice (Fig. 1A) following procedures described previously [4, 6]. The expression of IRF7 was verified by qRT-PCR and Western blot (Supplementary Fig. S1A, B). The leukemia cells in both models were GFP^+^CD3^-^CD19^-^CD11b^+^Gr-1^+^ (Supplementary Fig. S1C, D). The AML-IRF7^−/−^ mice exhibited higher levels of PB, SP and bone marrow leukemia cells since day 10 (Fig. 1B) and had shorter survival times than AML-WT mice (Fig. 1C). The sizes of spleens and livers on day 15 in the AML-IRF7^−/−^ group were larger than those in the AML-WT group (Fig. 1D, E). Furthermore, pathologic analysis showed that more infiltrating AML cells were detected in AML-IRF7^−/−^ mice than in AML-WT mice. Notably, intracerebral invasion of leukemia cells was detected in AML-IRF7^−/−^ mice (Fig. 1F, Supplementary Fig. S1E). The response of AML-IRF7^−/−^ cells to Ara-C was assessed by daily administration of Ara-C 4 times when PB AML cells reached approximately 6% in both groups. The PB leukemia cell levels were lower in the AML-IRF7^−/−^ group than in the AML-WT group at 24 and 48 h (Supplementary Fig. S1F, G). Disease progression was studied in the mice by the administration of Ara-C three times daily (Fig. 1G). Although it dropped to a similar level in the two groups on day 16, the level of PB leukemia cells recovered more quickly in AML-IRF7^−/−^ mice than in AML-WT mice (Fig. 1H). Moreover, the median survival time was prolonged by approximately 8 days in the AML-WT group versus approximately 5 days in the AML-IRF7^−/−^ group (Fig. 1I). These results suggest that loss of IRF7 in AML cells promotes disease progression.

### Characteristics of AML-IRF7^−/−^ cells

**Fig. 2:**
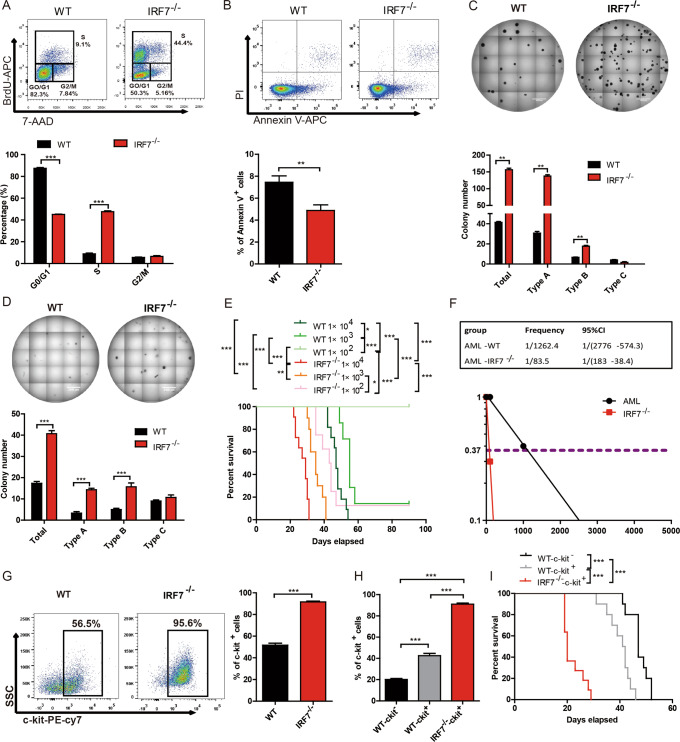
The characteristics of AML-IRF7^−/−^ cells. **A**–**D** The mice were intravenously injected with AML-WT or AML-IRF7^−/−^ cells on day 0 and sacrificed on day 15. **A** Representative results of BrdU incorporation experiments are shown (upper), and the percentages of G0/G1-, S- and G2/M-phase AML cells are plotted (lower). **B** Representative results of Annexin V and PI staining experiments are shown (upper), and the percentage of apoptotic AML cells is plotted (lower). **C**, **D** Primary (**C**) and secondary (**D**) colony formation experiments were performed by seeding 500 sorted AML cells per well into 24-well plates. Representative results are shown (upper), and colony numbers are plotted (lower). **E**, **F** AML-WT and AML-IRF7^−/−^ cells were sorted and transplanted into recipient mice in limiting-dilution series dosages. The survival of mice is shown in Kaplan–Meier curves (*n* = 10 for each group) (**E**), and the frequency of LSCs was estimated by using ELDA software (**F**). **G** Representative results of c-kit expression in AML cells are shown (left), and the percentage of c-kit^+^ AML cells is plotted (right). **H**, **I** AML-WT-c-kit^-^, AML-WT-c-kit^+^ and AML-IRF7^−/−^-c-kit^+^ cells were sorted and transplanted into mice. When mice suffered AML, the percentage of c-kit^+^ AML cells was analyzed (**H**), and the survival of mice is shown in Kaplan–Meier curves (*n* = 10 for each group) (**I**). Data are presented as mean ± S.E.M. Unpaired Student’s *t* test, one-way ANOVA tests and Kaplan–Meier estimates were used. **p < 0.01; ***p < 0.001.

The characteristics of AML-IRF7^−/−^ cells were first analyzed to determine the mechanism for the accelerated disease progression. No significant difference was detected in the homing potential of AML cells (Supplementary Fig. S1H). In contrast, in vivo BrdU experiments showed that more S phase cells but fewer G0/G1 phase cells were detected in AML-IRF7^−/−^ cells than in AML-WT cells (Fig. 2A). Furthermore, there were fewer apoptotic cells in AML-IRF7^−/−^ mice than in AML-WT mice (Fig. 2B).

Colony forming potential partly reflects the LSC level, which is vital for the initiation and relapse of leukemia. AML-IRF7^−/−^ cells formed more colonies than AML-WT cells in primary and secondary plating experiments (Fig. 2C, D). There are three types of colonies (Supplementary Fig. S2A) [4]. The AML-IRF7^−/−^ cells formed more type-A and type-B colonies than AML-WT cells (Fig. 2C, D). Limiting dilution transplantation experiments showed that 1 × 10^3^ AML cells caused 100% mortality in AML-IRF7^−/−^ mice but approximately 90% mortality in AML-WT mice, while 1 × 10^2^ AML cells caused approximately 90% mortality in AML-IRF7^−/−^ mice but no mortality in AML-WT mice (Fig. 2E). Furthermore, the LSC level of AML-IRF7^−/−^ cells was approximately 14-fold higher than that of AML-WT cells (Fig. 2F). These results indicate that AML-IRF7^−/−^ cells have more LSCs than AML-WT cells. C-kit is an important marker for primitive AML cells. The percentage of c-kit^+^ cells was approximately 50% in the AML-WT population, whereas it was over 90% in the AML-IRF7^−/−^ population (Fig. 2G). When AML cells were sorted based on c-kit expression and transplanted into secondary recipients, AML-WT-c-kit^-^ cells gave rise to approximately 25% c-kit^+^ cells, while AML-WT-c-kit^+^ and AML-IRF7^−/−^-c-kit^+^ cells gave rise to similar levels of c-kit^+^ cells as the first transplantation (Fig. 2H, Supplementary Fig. S2B). Furthermore, the AML-IRF7^−/−^-c-kit^+^ group had the shortest survival time (Fig. 2I). Hence, knockout of IRF7 promotes cell proliferation and increases LSC levels, which contribute to accelerated AML progression.

### Overexpression of IRF7 decreases cell proliferation and LSC levels

**Fig. 3:**
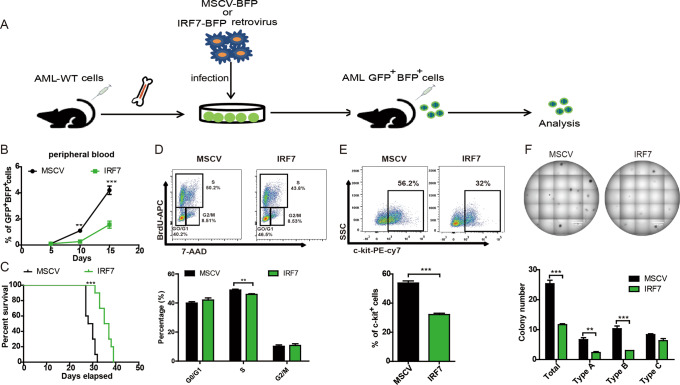
AML-IRF7 mice exhibit decreased disease progression. **A** Schematic overview of the development of the AML-MSCV and AML-IRF7 models. **B**, **C** Equal numbers of AML-MSCV and AML-IRF7 cells were transplanted into recipient mice. The percentage of peripheral blood GFP^+^BFP^+^ cells was monitored every 5 days (*n* = 4) (**B**), and the survival of mice is shown in Kaplan–Meier curves (*n* = 10) (**C**). **D**–**F** The mice were intravenously injected with AML-MSCV or AML-IRF7 cells and sacrificed at the middle stage of AML. **D** BrdU and 7-AAD staining was performed. Representative results are shown (upper), and the percentages of G0/G1, S, and G2/M phase AML cells are plotted (lower). **E** The expression of c-kit in AML cells was analyzed. Representative results are shown (upper), and the percentage of c-kit^+^ AML cells is plotted (lower). **F** Secondary colony formation experiments were performed by seeding 500 sorted AML cells per well into 24-well plates. Representative results are shown (upper), and the colony numbers are plotted (lower). Data are presented as mean ± S.E.M. Unpaired Student’s *t* test, one-way ANOVA tests and Kaplan–Meier estimates were used. **p < 0.01; ***p < 0.001.

AML-WT cells were infected with a blank or MSCV-mIRF7-BFP retroviral virus to construct AML models (Fig. 3A, Supplementary Fig. S3A). AML-MSCV and AML-IRF7 cells were GFP^+^BFP^+^, and AML-IRF7 cells expressed higher levels of IRF7 (Supplementary Fig. S3B, C). The PB AML cell level was lower in the AML-IRF7 group than in the AML-MSCV group on day 10 and day 15 (Fig. 3B). Markedly, the survival of AML-IRF7 mice was longer than that of AML-MSCV mice (Fig. 3C). In vivo BrdU experiments demonstrated that AML-IRF7 cells had decreased S phase cells (Fig. 3D). Furthermore, AML-IRF7 cells had decreased levels of c-kit^+^ cells (Fig. 3E). Moreover, AML-IRF7 cells formed fewer colonies (total, type-A and type-B) than AML-MSCV cells in primary and secondary plating experiment (Fig. 3F, Supplementary Fig. S3D). These results show opposite effects compared to those obtained from the AML-IRF7^−/−^ model, which further confirms that IRF7 negatively correlates with cell proliferation and LSC levels.

### Downregulation of TGIF1 contributes to malignant phenotypes in AML-IRF7^−/−^ cells

IRF7 activation results in the production of I-IFN [12]. It is necessary to determine whether exogenous administration of I-IFN can reverse the malignant phenotypes of AML-IRF7^−/−^ cells. Unexpectedly, this treatment did not alter the cell cycle, frequency of the c-kit^+^ population or colony forming potential of AML-IRF7^−/−^ cells, although it slightly increased apoptosis (Supplementary Fig. S4). Therefore, other mechanisms are likely responsible for the malignant phenotypes.

**Fig. 4:**
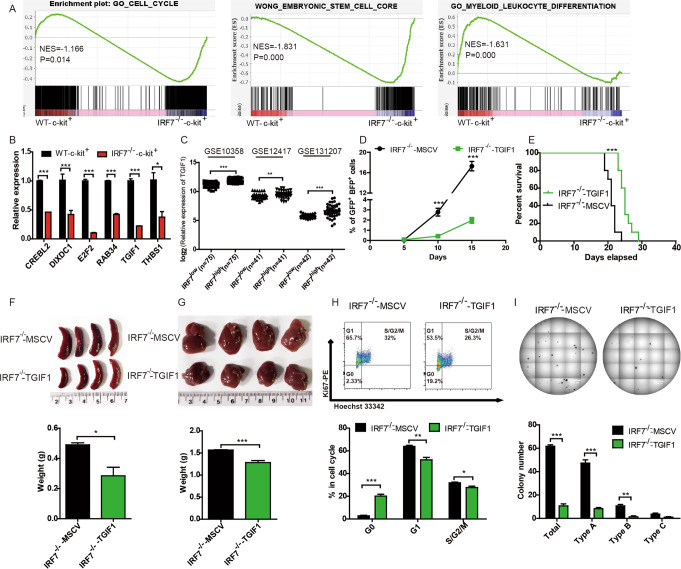
Downregulation of TGIF1 contributes to malignant phenotypes in AML-IRF7^−/−^ cells. **A** The enrichment of annotations of cell cycle, embryonic stem cell core, and myeloid leukocyte differentiation between AML-IRF7^−/−^-c-kit^+^ and AML-WT-c-kit^+^ cells by GSEA. **B** The expression of selected genes was analyzed by qRT-PCR. **C** The expression of TGIF1 between the IRF7^high^ group (top 30% cases) and IRF7^low^ group (bottom 30% cases) in the GSE10358, GSE12417, and GSE131207 datasets are shown. **D**, **E** Equal numbers of AML-IRF7^−/−^-MSCV and AML-IRF7^−/−^-TGIF1 cells were transplanted into recipient mice. **D** The percentage of peripheral blood AML cells was monitored every 5 days (*n* = 5). **E** The survival of mice is shown in Kaplan–Meier curves (*n* = 10 for each group). **F**–**I** The mice received transplantation of equal numbers of AML-IRF7^−/−^-MSCV and AML-IRF7^−/−^-TGIF1 cells on day 0 and were sacrificed on day 18. The sizes (upper) and weights (lower) of spleens (**F**) and livers (**G**) are shown. **H** GFP^+^BFP^+^ cells were stained with Ki67 and Hoechst 33342. Representative results are shown (upper), and the percentages of G0, G1, and S/G2/M phase AML cells are plotted (lower). **I** Secondary colony formation experiments were performed by seeding 500 sorted AML cells per well into 24-well plates. Representative results are shown (upper), and the colony numbers are plotted (lower). Data are presented as mean ± S.E.M. Unpaired Student’s *t* test, one-way ANOVA tests and Kaplan–Meier estimates were used. *p < 0.05; **p < 0.01; ***p < 0.001.

AML-WT-c-kit^-^, AML-WT-c-kit^+^, and AML-IRF7^−/−^-c-kit^+^ cells were sorted for microarray expression analysis. GSEA between AML-WT-c-kit^+^ and AML-IRF7^−/−^-c-kit^+^ cells demonstrated that the annotations, i.e., Cell cycle and embryonic stem cell core associated genes were enriched in AML-IRF7^−/−^-c-kit^+^ cells, whereas leukocyte differentiation associated genes were enriched in AML-WT-c-kit^+^ cells (Fig. 4A). These results imply that some DEGs may contribute to the increased proliferation and LSC level in AML-IRF7^−/−^ cells. A total of 2739 DEGs (|log2FC | ≥ 1 and FDR < 0.05), 1337 upregulated and 1402 downregulated, were detected between AML-IRF7^−/−^-c-kit^+^ and AML-WT-c-kit^+^ cells (Supplementary Fig. S5A). Among them, 137 genes met the conditions that their expression was lower in AML-IRF7^−/−^-c-kit^+^ cells than in both AML-WT-c-kit^+^ and AML-WT-c-kit^-^ cells. Furthermore, 46 genes within any of the gene sets of GSEA annotations (cell cycle and embryonic stem cell core associated genes) were enriched in AML-IRF7^−/−^-c-kit^+^ cells. Six of these genes were finally selected and verified by qRT-PCR (Fig. 4B). TGIF1 plays a negative role in AML progression [21]. The volcano plot shows that the lower expression of TGIF1 in AML-IRF7^−/−^-c-kit^+^ cells was significant (Supplementary Fig. S5B). Furthermore, a positive correlation between IRF7 and TGIF1 was detected in the human datasets GSE10358, GSE12417, and GSE131207 (Fig. 4C, Supplementary Fig. S5C, D). Moreover, downregulation of TGIF1 was detected when IRF7 was knocked down in THP1 and Kasumi-1 cells (Supplementary Fig. S5E, F). These results imply that downregulation of TGIF1 might mediate the proleukemic effects upon loss of IRF7.

To test this hypothesis, TGIF1 was overexpressed in AML-IRF7^−/−^ cells by MSCV-TGIF1-PGK-BFP retroviral infection. The AML-IRF7^−/−^-TGIF1 and AML-IRF7^−/−^-MSCV cells were GFP^+^BFP^+^ (Supplementary Fig. S6A). The expression of TGIF1 was verified by qRT-PCR (Supplementary Fig. S6B), and both AML cells were CD3^-^CD19^-^CD11b^+^Gr-1^+^ (Supplementary Fig. S6C, D). The AML-IRF7^−/−^-TGIF1 mice had lower levels of PB AML cells and a longer survival time than AML-IRF7^−/−^-MSCV mice after transplantation of an equal number of AML cells (Fig. 4D, E). The mice were sacrificed on day 15, and the sizes of the spleens and livers in the AML-IRF7^−/−^-TGIF1 group were smaller than those in the AML-IRF7^−/−^-MSCV group (Fig. 4F, G). In vivo Ki67 experiments demonstrated that AML-IRF7^−/−^-TGIF1 cells had increased levels of G0 phase cells but decreased levels of G1 and S/G2/M phase cells (Fig. 4H). No difference in apoptosis was detected between the two groups (Supplementary Fig. S6E). Colony forming experiments showed that AML-IRF7^-/-^-TGIF1 cells formed fewer colonies than AML-IRF7^−/−^-MSCV cells in primary and secondary plating experiment (Fig. 4I, Supplementary Fig. S6F).

These results demonstrate that overexpression of TGIF1 rescues the enhanced proliferation and the high LSC level caused by IRF7 deficiency, which suggests that TGIF1 mediates those effects.

### The role of IRF7-TGIF1 pathway in human AML cells

**Fig. 5:**
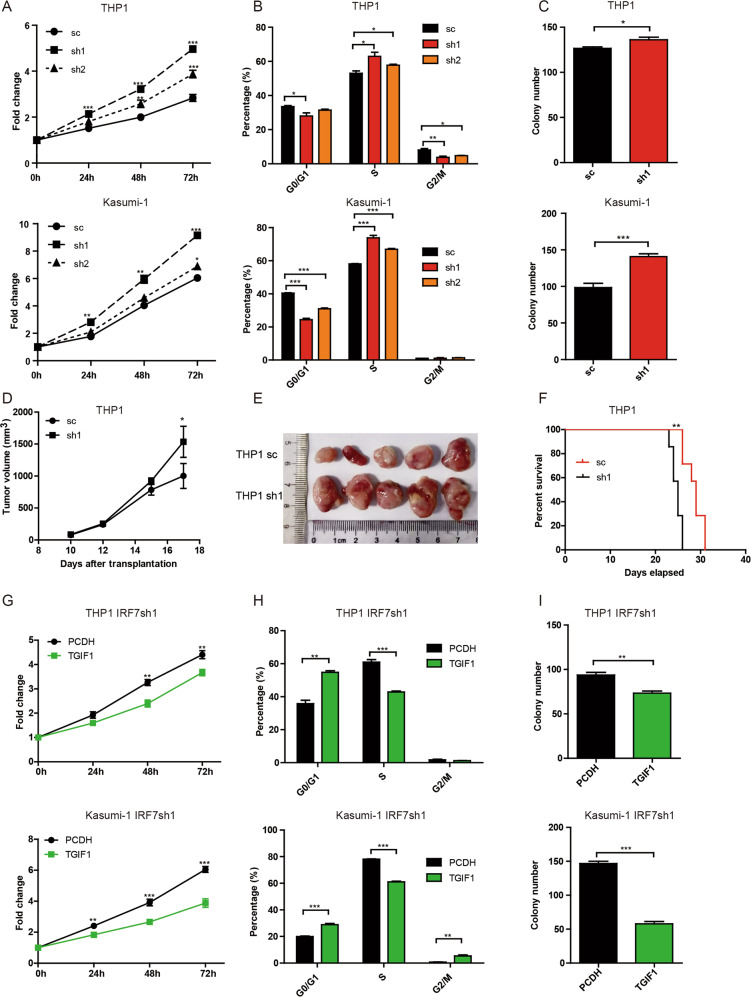
Knockdown of IRF7 promotes proliferation and increases LSC levels in human AML cell lines. **A**–**C** THP1 and Kasumi-1 cells were infected with pLV-IRF7sc, pLV-hIRF7sh1 and pLV-hIRF7sh2 lentiviruses. The GFP^+^ cells were sorted by flow cytometry. **A** Equal numbers of cells were seeded in 96-well plates for the MTS assay. The proliferation of cells is shown as the fold change against the OD value at 0 h. **B** The percentages of G0/G1, S, and G2/M phase cells assessed by BrdU and 7-AAD staining experiments are plotted. **C** Colony formation experiments were performed by seeding 500 sorted AML cells per well into 24-well plates. The colony numbers are plotted. **D**, **E** The nude mice were subcutaneously inoculated with an equal number of GFP^+^ cells on day 0 and sacrificed on day 17. The dynamic tumor volume is plotted (**D**) and the size of tumors are shown (**E**). **F** The NSG mice were intravenously transplanted with an equal number of GFP^+^ cells on day 0. The survival of mice is shown in Kaplan–Meier curves (*n* = 7 for each group). **G**–**I** THP1 IRF7sh1 and Kasumi-1 IRF7sh1 cells were infected with blank or PCDH-EF1α-hTGIF1-mRFP lentivirus. The GFP^+^RFP^+^ cells were sorted by flow cytometry. **G** Equal numbers of cells were added to 96-well plates for the MTS assay. The proliferation of cells is shown as the fold change against the OD value at 0 h. **H** GFP^+^RFP^+^ cells were stained with BrdU and 7-AAD. The percentage of G0/G1, S and G2/M phase cells is plotted. **I** Colony formation experiments were performed by seeding 500 AML cells per well into 24-well plates. The colony numbers are plotted. Data are presented as mean ± S.E.M. Unpaired Student’s *t* test and one-way ANOVA tests were used. *p < 0.05; **p < 0.01; ***p < 0.001.

THP1 and Kasumi-1 cells were used to test whether the IRF7-TGIF1 pathway affects the malignant phenotypes of human AML cells. First, the expression of IRF7 was knocked down in THP1 and Kasumi-1 cells by lentiviral infection. The successfully constructed cell lines were GFP^+^ (Supplementary Fig. S7A, B) and the expression of IRF7 was verified by qRT-PCR (Supplementary Fig. S7C, D). MTS (Fig. 5A), BrdU (Fig. 5B, Supplementary Fig. S7E, F) and Ki67 experiments (Supplementary Fig. S7G, H) demonstrated that knockdown of IRF7 promoted cell proliferation. Furthermore, THP1sh1 and Kasumi-1sh1 cells formed more colonies than their respective controls (Fig. 5C, Supplementary Fig. S7I, J). Moreover, nude mice xenograft experiments demonstrated that THP1sh1 cells formed larger tumors than control cells in vivo (Fig. 5D, E). Xenograft transplantation experiments in NSG mice also demonstrated that THP1sh1 group had shorter survival time than THP1sc group. These results suggest that knockdown of IRF7 also plays an adverse role in human AML cells.

Then, TGIF1 was overexpressed in THP1sh1 and Kasumi-1sh1 cells by lentiviral infection. After sorting, the successfully constructed cell lines were GFP^+^RFP^+^ (Supplementary Fig. S8A, B), and the expression of TGIF1 was verified by qRT-PCR (Supplementary Fig. S8C, D). MTS (Fig. 5G), BrdU (Fig. 5H, Supplementary Fig. S8E, F) and Ki67 (Supplementary Fig. S8G, H) experiments demonstrated that overexpression of TGIF1 in THP1sh1 and Kasumi-1sh1 cells promoted cell proliferation. In addition, overexpression of TGIF1 in THP1sh1 and Kasumi-1sh1 cells formed fewer colonies than their respective controls (Fig. 5I, Supplementary Fig. S8I, J). These results suggest that overexpression of TGIF1 rescues the adverse effects of IRF7 deficiency in human AML cells.

### Blocking the VCAM1-VLA-4 axis delays disease progression and attenuates intracerebral invasion of AML-IRF7^−/−^ cells

**Fig. 6:**
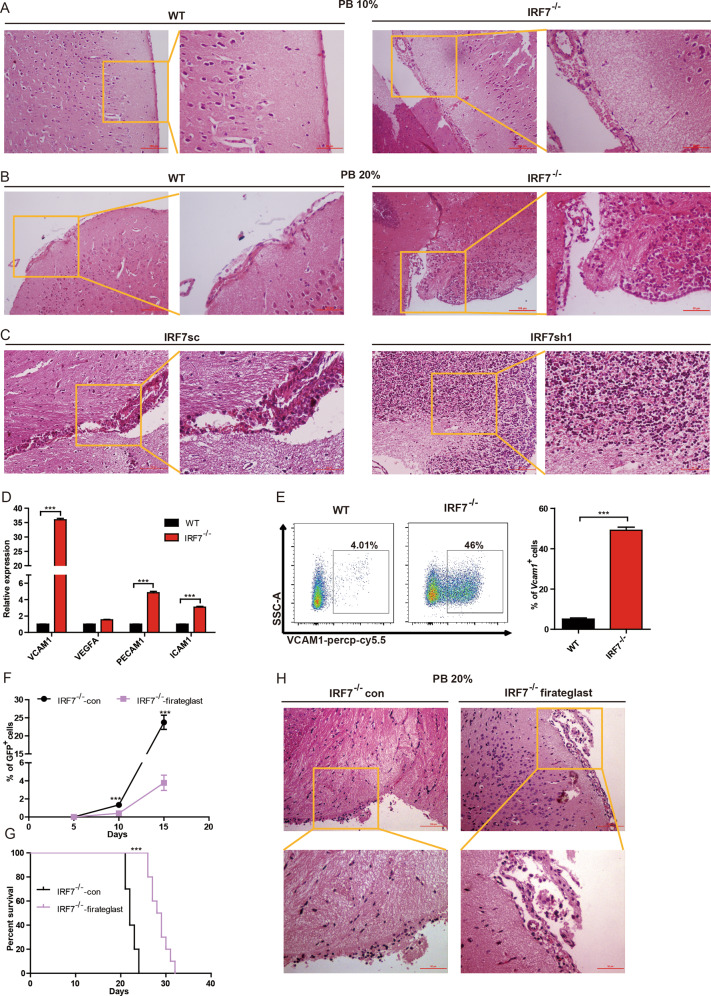
Blocking VCAM1-VLA-4 axis delays disease progression and attenuates intracerebral invasion in AML-IRF7^−/−^ mice. **A**, **B** An equal number of AML-WT or AML-IRF7^−/−^ cells were transplanted into the mice on day 0 and the mice were sacrificed when peripheral blood GFP^+^ cells reached 10% (**A**) and 20% (**B**). Representative results of HE-stained brain sections are shown. Scale bars, 100 or 50 μm. **C** An equal number of THP1sc or THP1sh1 cells were transplanted into the NSG mice on day 0 and the mice were sacrificed on day 25. Representative results of HE-stained brain sections are shown. Scale bars, 100 or 50 μm. **D** The expression of genes was analyzed by qRT-PCR. **E** The expression of VCAM1 in AML cells was analyzed by flow cytometry. Representative results are shown (left), and the percentage of VCAM1^+^ cells is plotted (right). **F**, **G** The mice were intravenously injected with an equal number of AML-IRF7^−/−^ cells followed by daily administration with or without firategrast. The percentage of peripheral blood AML cells was monitored every 5 days (*n* = 5) (**F**), and the survival of mice is shown in Kaplan–Meier curves (*n* = 10) (**G**). **H** The mice were intravenously injected with an equal number of AML-IRF7^−/−^ cells followed by daily administration of firategrast or not. The mice were sacrificed when PB GFP^+^ cells reached 20%. Representative results of HE-stained brain sections are shown. Scale bars, 100 or 50 μm. Data are presented as mean ± S.E.M. Unpaired Student’s *t* test, one-way ANOVA tests and Kaplan–Meier estimates were used. ***p < 0.001.

An interesting observation from this study is that AML-IRF7^−/−^ cells cross the BBB more easily and cause intracerebral invasion, which is an adverse factor in AML. To further confirm this phenomenon, AML-WT and AML-IRF7^−/−^ mice were sacrificed when the PB AML cells were 10% or 20%. All AML-IRF7^−/−^ mice but no AML-WT mice suffered submeningeal infiltration of AML cells at the 10% stage (Fig. 6A). At the 20% stage, intracerebral invasion (4/5) and submeningeal infiltration (5/5) were detected in AML-IRF7^-/-^ mice, whereas only submeningeal infiltration (5/5) was detected in AML-WT mice (Fig. 6B). However, no significant difference was detected in the spleen, liver, or bone marrow at the 20% stage (Supplementary Fig. S9A). Furthermore, xenograft transplantation experiments in NSG mice also demonstrated that knockdown of IRF7 in THP1 cells promoted the degree of CNS infiltration on day 25 (Fig. 6C).

Adhesion molecules and chemokine receptors are vital for malignant cells to cross the BBB [26]. Among them, VLA-4 in lymphocytic leukemia cells is a key molecule in this process [27]. However, a dramatic decrease in Integrin α4 and a slight increase in Integrin β1 were detected in AML-IRF7^−/−^ cells (Supplementary Fig. S9B). In contrast, microarray results demonstrated that VCAM1 is critical since its expression was the highest in AML-IRF7^−/−^-c-kit^+^ cells but the lowest in AML-WT-c-kit^-^ cells (Supplementary Fig. S9C). The expression of VCAM1, VEGFA, PECAM1, and ICAM1, which promote leukemia cells to cross the BBB [26, 27], was studied by RT-PCR. The highest expression increase was observed for VCAM1 (Fig. 6D). Flow cytometry results further demonstrated that AML-IRF7^−/−^ cells expressed higher levels of VCAM1 than AML-WT cells (Fig. 6E). It was reported that VCAM1^+^ cells exhibited more stem cell characteristics in neural stem cell populations [29], which implies that stem cells may express high levels of VCAM1. In fact, a positive correlation between LSC level and VCAM1 expression was observed in the datasets GSE92969 and GSE115549 [4, 6]. Notably, VCAM1^+^ cells were only detected in the c-kit^+^ population of AML-WT cells (Supplementary Fig. S9D), while the positive rate and MFI of VCAM1 were higher in the c-kit^high^ population of AML-IRF7^−/−^ cells than in either the c-kit^low^ or the c-kit^-^ population (Supplementary Fig. S9E). Therefore, the LSC levels in AML may be positively correlated with VCAM1 expression. Furthermore, the expression of VLA-4 was reported in endothelial cells [30]. Moreover, RT-PCR results showed that both subunits of VLA-4 were expressed in a brain microvascular endothelial cell line, Bend.3, and confocal microscopy further confirmed the expression of integrin α4 at the protein level (Supplementary Fig. S9F, G). Hence, high levels of VCAM1 in AML cells may contribute to this pathologic process.

Firategrast, an inhibitor of VLA-4, was used to block this axis to further explore the mechanism. Firategrast had no effect on the proliferation, apoptosis, and colony forming potential of AML-IRF7^−/−^ cells (Supplementary Fig. S10A-D). However, PB AML cell levels were lower in mice treated with firategrast beginning on day 10 (Fig. 6F). Furthermore, firategrast treatment prolonged the survival of AML-IRF7^−/−^ mice (Fig. 6G). At the 20% stage, treatment had little effect on the distribution of AML-IRF7^−/−^ cells in the spleen, bone marrow, and liver (Supplementary Fig. S10E). Whereas treatment reduced the degree of CNS infiltration, i.e., Intracerebral invasion (1/5) and submeningeal infiltration (5/5) were detected in the treatment group, while intracerebral invasion (5/5) was detected in the control group (Fig. 6H).

Collectively, the VCAM1-VLA-4 axis is crucial for IRF7 deletion-related intracerebral invasion; blocking this axis delays AML progression and attenuates intracerebral invasion.

## Discussion

Much attention is given to the mechanisms that IRFs modulate tumor immunomicroenvironment [10, 11]. IRF7 has been implicated in tumor progression and microenvironment-related mechanisms have been proposed [16, 31], while opposite intrinsic effects on tumor progression were reported in different tumors [17, 18]. In hematopoiesis, IRF7 promotes the proliferation of HSCs under stress conditions [32]. Downregulation of IRF7 was detected in some AML cases with specific genetic abnormalities from Bloodspot. Nevertheless, the role of IRF7 in leukemia has not been established. In this study, we focused on the intrinsic effects of IRF7 on AML cells showing that knockout of IRF7 exerted multifaceted effects to accelerate disease progression while blocking VCAM1-VLA-4 axis delayed disease progression and attenuated intracerebral invasion. CNSL is widely studied in lymphocytic leukemia [33] but limited in myeloid leukemia [34, 35]. Hence, our study not only contributes to a deep understanding of the pathologic role of IRF7 in AML progression but also provides a potential strategy to control myeloid CNSL.

High proliferative potential and LSC/tumor stem cell (TSC) level are two well-established adverse factors in malignancies. They seem to be mutually exclusive since LSCs/TSCs are considered quiescent cells [36, 37]. However, malignant cells are composed of heterogeneous populations, and some key molecules may participate in the maintenance of LSCs/TSCs and the proliferation of non-LSCs. Therefore, malignancies with both characteristics are not rare [4, 6]. Since these adverse characteristics are always associated with poor prognosis [38, 39], efforts are made to elucidate the mechanisms. I-IFN is a potential candidate mediating the effects since there is a positive feedback between IRF7 and I-IFN [40], and I-IFN has antitumor activity [41]. However, exogenous administration of I-IFN failed to reverse the effects. Since I-IFN is also positively regulated by other IRFs, including IRF1, IRF3, IRF5, and IRF8 [42], loss of IRF7 may have a limited effect on the production of I-IFN. Hence, I-IFN may not mediate the adverse effects caused by IRF7 deficiency. TGIF1, a suppressor of TGF-β signaling [19], is another candidate since we found a positive correlation between IRF7 and TGIF1 in mouse models, human cell lines, and human datasets. In hematopoiesis, loss of TGIF1 promotes the maintenance of normal HSCs [20], and leads to enhanced proliferation, increased LSC frequency, accelerated disease progression in AML [22]. We demonstrated that overexpression of TGIF1 in IRF7^−/−^ AML cells and human AML cells rescued both adverse phenotypes caused by loss of IRF7, suggesting that TGIF1 mediates those effects in AML progression. The direct interaction between IRF7 and TGIF1 has not been elucidated. However, IRF7 can be coimmunoprecipitated with Smad3 [43], which also interact with TGIF1 [19]. Furthermore, the deletion of IRF7 hinders the upregulation of genes in the MAPK pathway [44], which is upstream of TGIF1 phosphorylation [45]. Therefore, although a link between IRF7 and TGIF1 has been observed and the IRF7-TGIF1 axis has been implicated, the molecular mechanism requires further investigation.

CNSL is associated with poor prognosis in both lymphocytic and myeloid leukemia [46, 47]. CNSL-AML cases have higher relapse rate [48]. Intrathecal chemotherapy may cause neurocognitive deficits and secondary malignancies [9]. IRF7^−/−^ AML is a good myeloid CNSL mouse model to explore therapeutic strategies. We found that the VCAM1-VLA-4 axis, specifically VCAM1(AML cells)-VLA-4 (endothelial cells), was crucial in this pathologic process since a dramatic increase of VCAM1 but not VLA-4 was detected in AML-IRF7^−/−^ cells while endothelial cells expressed VLA-4. Notably, the mechanism is different from those reported in the literature, i.e., VLA-4 (leukocytes or lymphoblastic leukemia cells)-VCAM1 (endothelial cells) [26]. Other axis, such as ICAM-1-LFA-1 [27], and molecules, such as PECAM1 [27], CCLs, CXCLs, and MMPs [26], may also contribute to this process. Nevertheless, they are less crucial since low degree increase or no increase was detected in AML-IRF7^−/−^ cells. Importantly, blocking VCAM1-VLA-4 axis attenuated intracerebral invasion and prolonged the survival of AML mice, which might be a potential strategy for the treatment of myeloid CNSL. The mechanism for the upregulation of VCAM1 in AML-IRF7^−/−^ cells has not been established. However, it is interesting that the positive correlation between LSC level and VCAM1 expression was also reported in some AML models [4, 6]. Furthermore, a positive correlation between LSC level and intramedullary infiltration was reported in some AML models [49]. Further work is needed to elucidate the mechanism.

Collectively, we demonstrate that loss of IRF7 in AML cells accelerates disease progression by simultaneously promoting cell proliferation, elevating LSC levels, and causing intracerebral invasion. Downregulation of TGIF1 in AML cells accounts for enhanced proliferation and increased LSC levels. Upregulation of VCAM1 in AML cells accounts for intracerebral invasion. Therefore, IRF7 is an intrinsic suppressive factor in AML progression and blocking VCAM1-VLA-4 axis may be a potential strategy for controlling myeloid CNSL.

## Material and methods

### Cell lines

In knockdown experiments, THP1 and Kasumi-1 cells were infected with pLV-hIRF7sc, pLV-hIRF7sh1, and pLV-hIRF7sh2 lentiviruses. The GFP^+^ cells were sorted 2 days later and designated THP1sc, THP1sh1, THP1sh2, Kasumi-1sc, Kasumi-1sh1, and Kasumi-1sh2.

To explore the effect of TGIF1, THP1sh1 and Kasumi-1sh1 cells were infected with blank or PCDH-EF1α-hTGIF1-mRFP lentivirus. The GFP^+^RFP^+^ cells were sorted 2 days later and named THP1-IRF7sh1-PCDH, THP1-IRF7sh1-TGIF1, Kasumi-1-IRF7sh1-PCDH and Kasumi-1-IRF7sh1-TGIF1.

### Mice

C57BL/6 J, NOD/LtSz-scid IL2rγ^null^ (NSG) and nude mice were provided by the Animal Center of the Institute of Hematology and Blood Diseases Hospital, CAMS & PUMC. IRF7^−/−^ mice (C57BL/6 J background) were constructed by replacing the exon 2 and 3 of Irf7 gene with a PGK-beta-geo cassette [50]. They were obtained from the Institute of Zoology, CAMS & PUMC. Mice were maintained in specific pathogen-free (SPF)-certified facilities. Seven- to eight-week-old mice were used in all experiments, which were approved by the Animal Care and Use Committee at the institution.

### Mouse AML models

The establishment of the MLL-AF9-induced AML model was previously described [4, 6]. Briefly, c-kit^+^ cells from WT or IRF7^−/−^ mice were infected with MSCV-MLL-AF9-GFP retrovirus before transplantation into mice by intravenous tail injection (Fig. 1A). The cells were AML-WT and AML-IRF7^−/−^ cells, while the mice were AML-WT and AML-IRF7^−/−^ mice.

In the AML model overexpressing IRF7, GFP^+^ AML-WT cells were infected with MSCV-mIRF7-PGK-BFP or MSCV-PGK-BFP retrovirus. GFP^+^BFP^+^ cells were sorted and transplanted into mice. The cells were AML-MSCV and AML-IRF7 cells, while the mice were AML-MSCV and AML-IRF7 mice.

In the AML model overexpressing TGIF1, GFP^+^ AML-IRF7^−/−^ cells were infected with MSCV-PGK-BFP and MSCV-mTGIF1-PGK-BFP. GFP^+^BFP^+^ cells were sorted and transplanted into mice. The cells were AML-IRF7^−/−^-MSCV and AML-IRF7^−/−^-TGIF1 cells, while the mice were AML-IRF7^−/−^-MSCV and AML-IRF7^−/−^-TGIF1 mice.

### Limiting-dilution transplantation

GFP^+^ AML cells were sorted from AML-WT or AML-IRF7^−/−^ mice and transplanted into recipient mice in limiting-dilution series dosages. The survival of the mice was recorded. The frequency of LSCs was calculated in terms of Poisson statistics using extreme limiting dilution analysis (ELDA) software.

### Firategrast treatment

The VLA-4 inhibitor firategrast (MedChemExpress, USA) was used to block the VCAM1-VLA-4 interaction [51]. Mice were transplanted with 1 × 10^5^ AML-IRF7^−/−^ cells on day 0. The mice were administered firategrast (20 mg/kg/day) daily by oral gavage from day 0 to the end of their lives. The PB GFP^+^ cells were monitored on days 5, 10, and 15. The survival of mice was recorded. For pathologic analysis of tissue sections, the mice were sacrificed when PB GFP^+^ cells reached approximately 20%. Standard hematoxylin-eosin (HE) staining was performed.

### Gene expression microarray

The AML-WT-c-kit^-^, AML-WT-c-kit^+^, and AML-IRF7^−/−^-c-kit^+^ cells were sorted by flow cytometry. Microarrays were carried out by Shanghai Majorbio Biopharm Technology (China) following standard protocols. The data are available in the National Center for Biotechnology Information Gene Expression Omnibus database under accession number GSE178560. Data for all genes were subjected to gene set enrichment analyses (GSEAs) to investigate the biological functions or pathways. Standard analyses were also performed on the online Majorbio Cloud Platform. Fold change (FC) ≥ 2.0 and *p* value < 0.05 were used as the cutoff for screening the differentially expressed genes (DEGs). The DEGs between AML-IRF7^−/−^-c-kit^+^ and AML-WT-c-kit^+^ cells are shown in a volcano plot.

### Statistical analysis

All experiments were repeated two to three times in accordance with the indicated numbers of mice. The sample size of each experiment is determined based on experience or previously published papers. The investigator was not blinded during experiment or evaluating the result. The SPSS 16.0 software package (SPSS, IL) and GraphPad Prism 5.0 (GraphPad Software, CA) were used. Shapiro-Wilks method was used for distribution test. When parameters followed Gaussian distribution, unpaired Student’s *t* test was used for comparisons between two groups, whereas one-way ANOVA was used for comparisons among multiple groups. Kaplan–Meier estimates were used for survival curves. ELDA was used for the limiting-dilution transplantation experiments. *P* < 0.05 was considered statistically significant. Statistically, we have compared similar variances between the groups as well.

Additional experimental procedures are provided in the Supplementary Experimental Methods.

## Supplementary information

supplemental figure

supplemental materials
