# Supplementary material for: Loss of IRF7 accelerates acute myeloid leukemia progression and induces VCAM1-VLA-4 mediated intracerebral invasion
Source: Oncogene. 2022 Mar 7;41(16):2303–14. doi: 10.1038/s41388-022-02233-w (PMC9010288; doi:10.1038/s41388-022-02233-w)
Supplement: Supplementary file 1 — supplemental figure [file 41388_2022_2233_MOESM1_ESM.pdf]

Wang H, et al. Figure S1

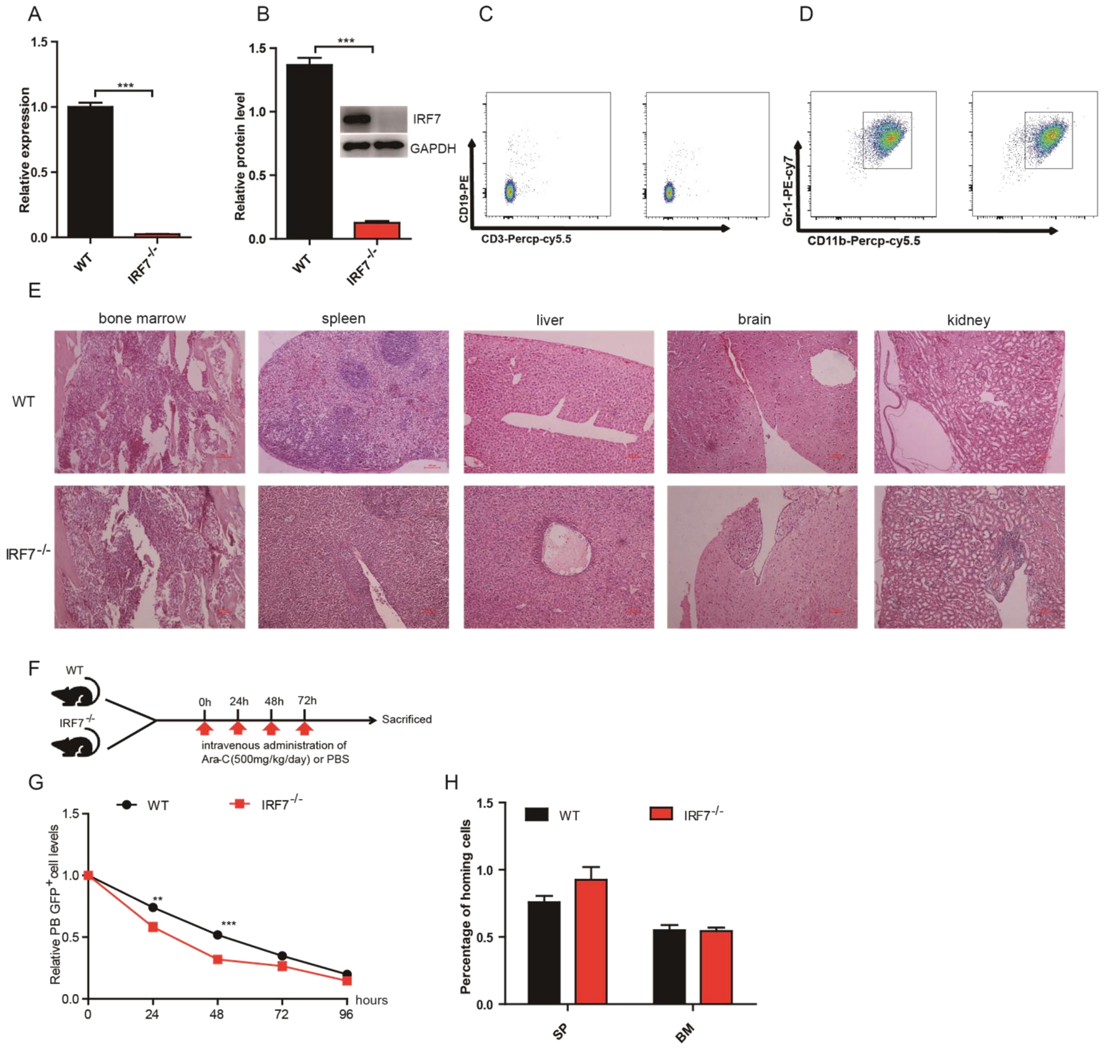

Fig. S1

Establishment of the AML-IRF7$^{-/-}$ model and treatment with Ara-C.

(A-E) The mice received a transplantation of 1×10^6 AML-WT or AML-IRF7$^{-/-}$ cells on day 0 and were sacrificed on day 15. The expression of IRF7 in sorted AML cells was analyzed by qRT-PCR (A) and Western blot (B). (C, D) Surface markers (GR-1, CD11b, CD3 and B220) of AML cells were analyzed by flow cytometry. (E) Representative HE-stained sections of bone marrow, spleen, liver, brain and kidney are shown. Scale bars, 200μm. (F) Schematic overview of the experiments with multiple-dose administrations of Ara-C to AML mice. (G) The relative PB GFP$^+$ cell levels were monitored. (H) In homing experiments, 1×10^7 AML-WT or AML-IRF7$^{-/-}$ cells were transplanted into mice. The percentage of GFP$^+$ cells in spleen and bone marrow was detected after 16 hours. Data are presented as mean ± S.E.M. **$p < 0.01$; *** $p < 0.001$.

Wang H, et al. Figure S2

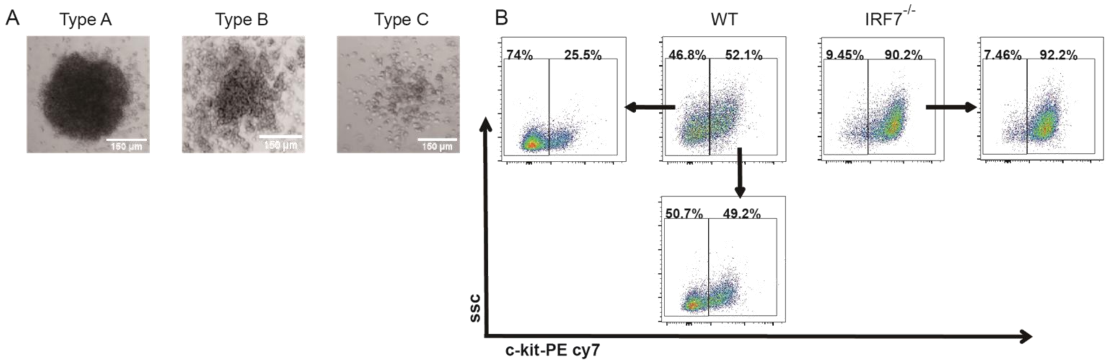

Fig. S2

Colonies, c-kit expression in AML-WT and AML-IRF7$^{-/-}$ cells.

(A) Typical type-A, type-B and type-C colonies observed in this study are shown. (B) AML-WT-c-kit^{-} and AML-WT-c-kit^{+} cells were sorted from AML-WT mice, while AML-IRF7$^{-/-}$-c-kit^{+} cells were sorted from AML-IRF7$^{-/-}$ mice. Then, they were transplanted into second recipients. At the middle stage of leukemia, the composition of the AML population based on c-kit expression was analyzed by flow cytometry. The representative results are shown.

Wang H, et al. Figure S3

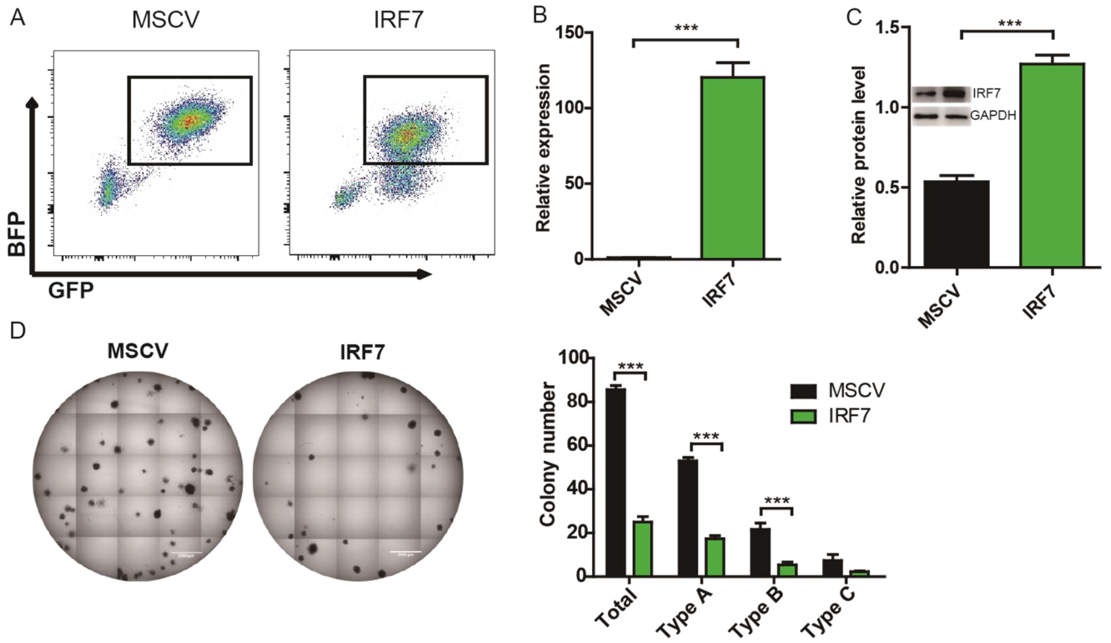

Fig. S3

Establishment of a mouse AML model overexpressing IRF7.

(A) AML-WT cells were infected with a blank or MSCV-mIRF7-BFP retroviral virus. GFP^+BFP^+ cells were sorted by flow cytometry for further study. The expression of IRF7 was analyzed by qRT-PCR (B) and Western blot (C). (D) Primary colony formation experiments were performed by seeding 500 sorted AML cells per well into 24-well plates. Representative results are shown (left), and colony numbers are plotted (right). Data are presented as mean ± S.E.M. *** $p < 0.001$.

Wang H, et al. Figure S4

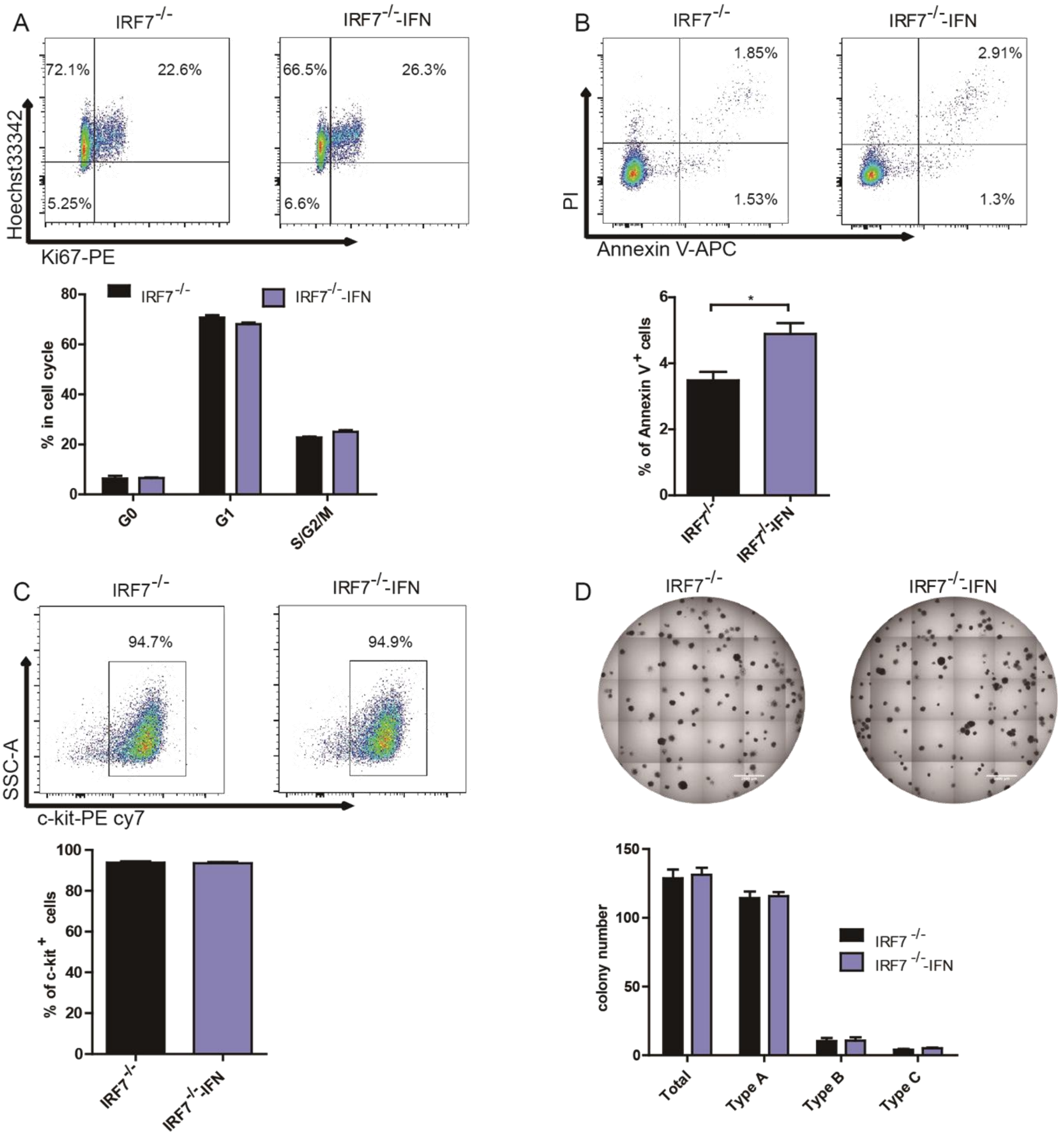

Fig. S4

The effect of I-IFN on AML-IRF7$^{-/-}$ cells.

Mice were intravenously injected AML-IRF7$^{-/-}$ cells. At the middle stage of leukemia, mice were intraperitoneally injected with IFNα and IFNβ and sacrificed on the second day. (A) GFP^{+} leukemia cells were stained with Ki67 and Hoechst 33342. Representative flow cytometry results are shown (upper), and the percentage of G0/G1, S and G2/M phase AML cells is plotted (lower). (B) Representative results of Annexin V and PI staining experiments are shown (upper), and the percentage of apoptotic AML cells is plotted (lower). (C) Representative results of c-kit expression in AML cells are shown (upper), and the percentage of c-kit^{+} AML

cells is plotted (lower). (D) Colony formation experiments were performed by seeding 500 sorted AML cells per well into 24-well plates. Representative results of the high-content analysis system are shown (upper), and the colony numbers are plotted (lower). Data are presented as mean ± S.E.M. *$p < 0.05$.

Wang H, et al. Figure S5

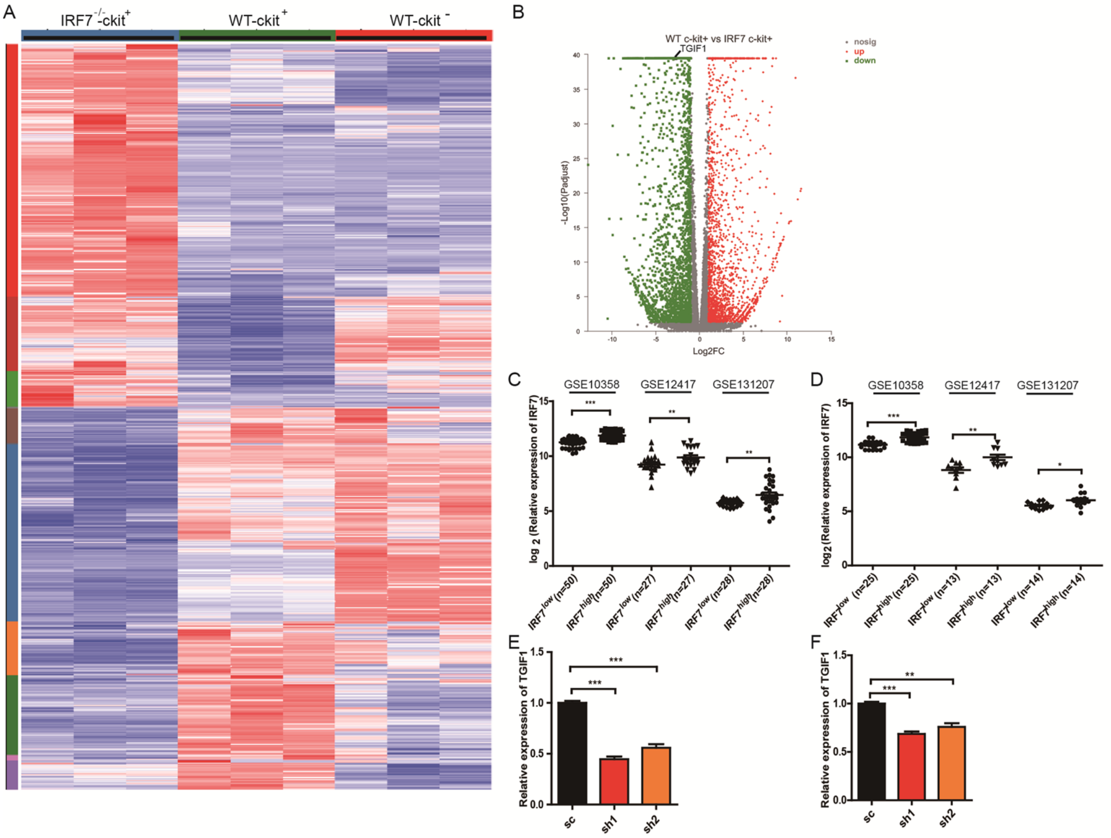

Fig. S5

Screening key molecules mediating pro-leukemic effects in AML-IRF7$^{-/-}$ cells.

(A) The expression profile of the genes, which are the DEGs between AML-WT-c-kit^{+} and AML-IRF7$^{-/-}$-c-kit^{+} cells, in AML-WT-c-kit^{-}, AML-WT-c-kit^{+} and AML-IRF7$^{-/-}$-c-kit^{+} cells, was subjected to hierarchical clustering analysis. (B) Volcano plot of AML-WT-c-kit^{+} and AML-IRF7$^{-/-}$-c-kit^{+} is shown, and TGIF1 is indicated. (C) The expression of TGIF1 in the IRF7^{high} group (top 20% cases) and IRF7^{low} group (bottom 20% cases) in datasets GSE10358, GSE12417 and GSE131207 is shown. (D) The expression of TGIF1 in the IRF7^{high} group (top 10% cases) and IRF7^{low} group (bottom 10% cases) in datasets GSE10358, GSE12417 and GSE13120 is shown. (E) THP1 and Kasumi-1 cells were infected with pLV-IRF7sc, pLV-hIRF7sh1 and pLV-hIRF7sh2 lentiviruses. The relative expression of TGIF1 in GFP^{+} cells was determined by qRT-PCR. Data are presented as mean ± S.E.M. *$p < 0.05$; **$p < 0.01$; *** $p < 0.001$.

Wang H, et al. Figure S6

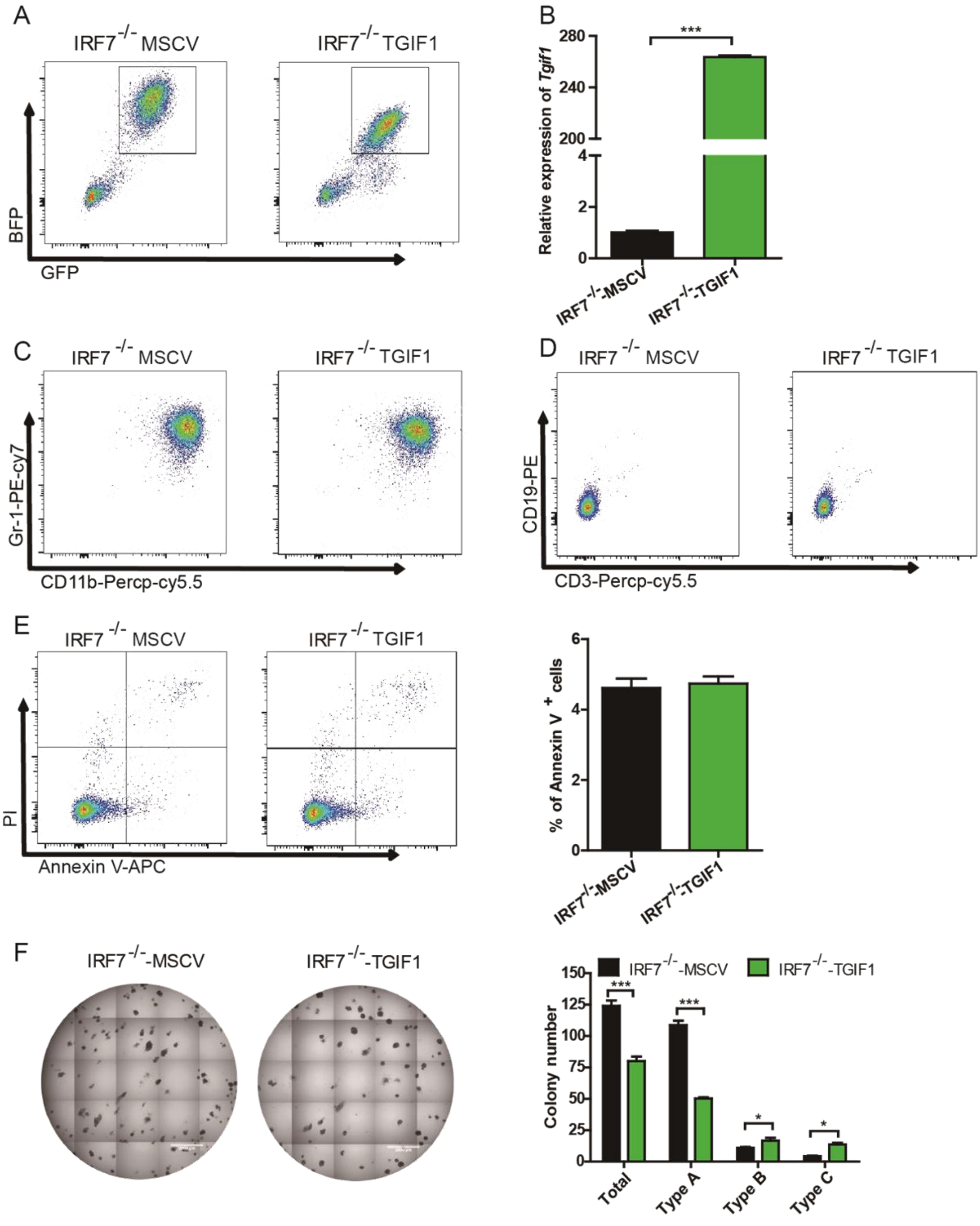

Fig. S6

Overexpression of TGIF1 in mouse AML-IRF7$^{-/-}$ cells.

(A) AML-IRF7$^{-/-}$ cells were infected with blank or MSCV-TGIF1-PGK-BFP retrovirus. GFP^{+}BFP^{+} AML-IRF7$^{-/-}$-MSCV and AML-IRF7$^{-/-}$-TGIF1 cells were sorted for further use. (B) The expression of TGIF1 was analyzed by qRT-PCR. (C, D) The surface markers (GR-1, CD11b, CD3 and B220) of AML-IRF7$^{-/-}$-MSCV and AML-IRF7$^{-/-}$-TGIF1 cells were analyzed by flow cytometry. (E) Representative results of Annexin V and PI staining experiments are shown

(left), and the percentage of apoptotic AML cells is plotted (right). (F) Primary colony formation experiments were performed by seeding 500 sorted AML cells per well into 24-well plates. Representative results are shown (left), and colony numbers are plotted (right). Data are presented as mean ± S.E.M. *** $p < 0.001$.

Wang H, et al. Figure S7

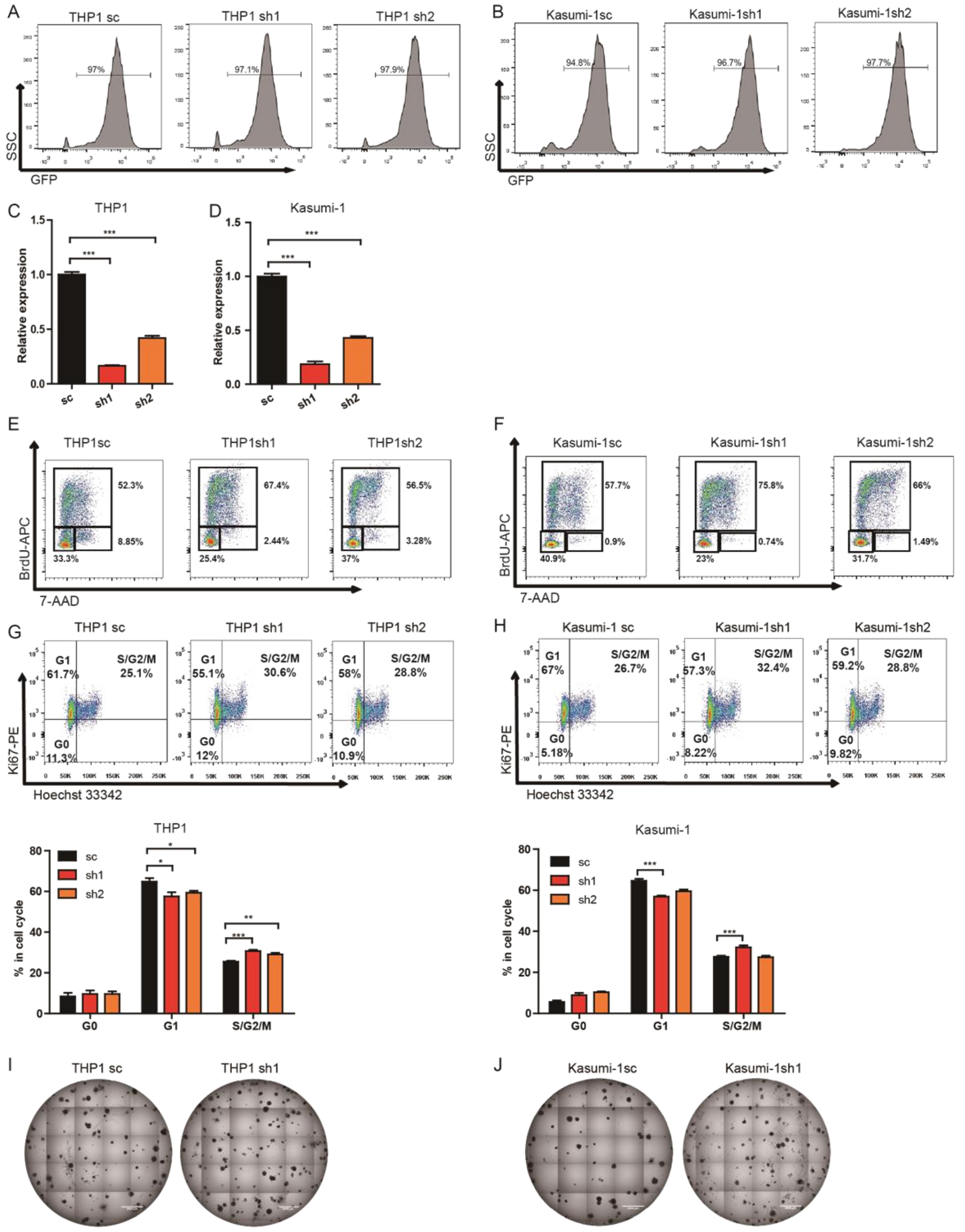

Fig. S7

Knockdown of IRF7 in THP1 and Kasumi-1 cells.

THP1 and Kasumi-1 cells were infected with pLV-hIRF7sc, pLV-hIRF7sh1 and pLV-hIRF7sh2 lentiviruses. (A, B) The percentage of GFP^+ cells was determined by flow cytometry analysis. (C, D) The expression of IRF7 was analyzed by qRT-PCR. (E, F) GFP^+ cells were stained with BrdU and 7-AAD and analyzed by flow cytometry. The representative results are shown. (G, H)

GFP$^+$ cells were stained with Ki67 and Hoechst 33342 and analyzed by flow cytometry. The representative results are shown (upper) and the percentages of G0, G1 and S/G2/M phase AML cells are plotted (lower). (I, J) Five hundred GFP$^+$ cells were sorted and seeded into 24-well plates. Representative results of the high-content analysis system are shown. Data are presented as mean ± S.E.M. *** $p < 0.001$.

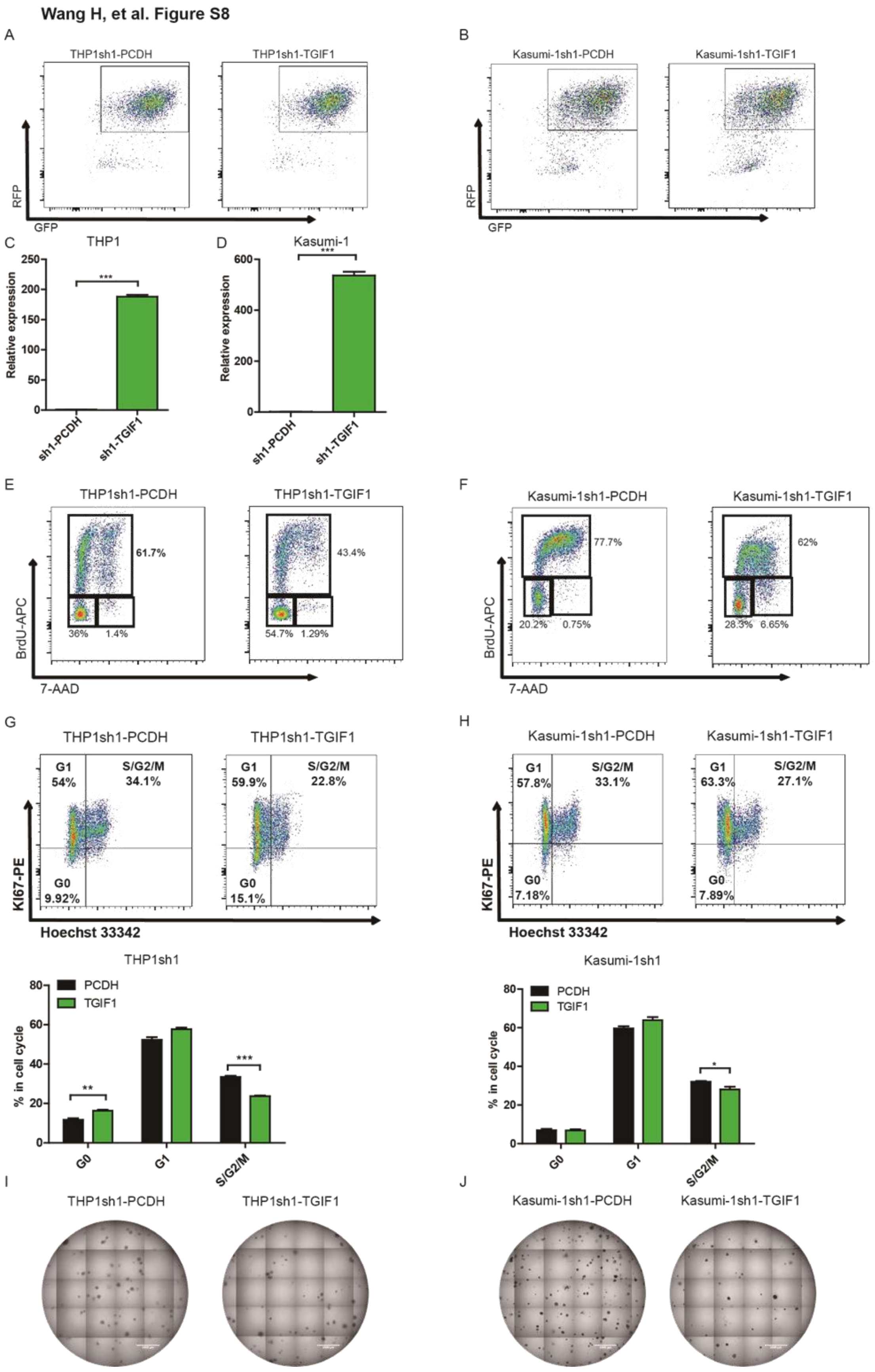

Fig. S8

Overexpression of TGIF1 in THP1IRF7sh1 and Kasumi-1IRF7sh1 cells.

THP1IRF7sh1 and Kasumi-1IRF7sh1 cells were infected with blank or PCDH-EF1α-hTGIF1-mRFP lentivirus. (A, B) GFP^+RFP^+ cells were sorted by flow cytometry. (C, D) The expression of TGIF1 was analyzed by qRT-PCR. (E, F) GFP^+RFP^+ cells were stained with BrdU and 7-AAD. Representative results are shown. (G, H) GFP^+RFP^+ cells were stained with Ki67 and Hoechst 33342 and analyzed by flow cytometry. The representative results are shown (upper) and the percentages of G0, G1 and S/G2/M phase AML cells are plotted (lower). (I, J) Colony formation experiments were performed by seeding 500 AML cells per well into 24-well plates. Representative results of the high-content analysis system are shown. Data are presented as mean ± S.E.M. *** $p < 0.001$.

Wang H, et al. Figure S9

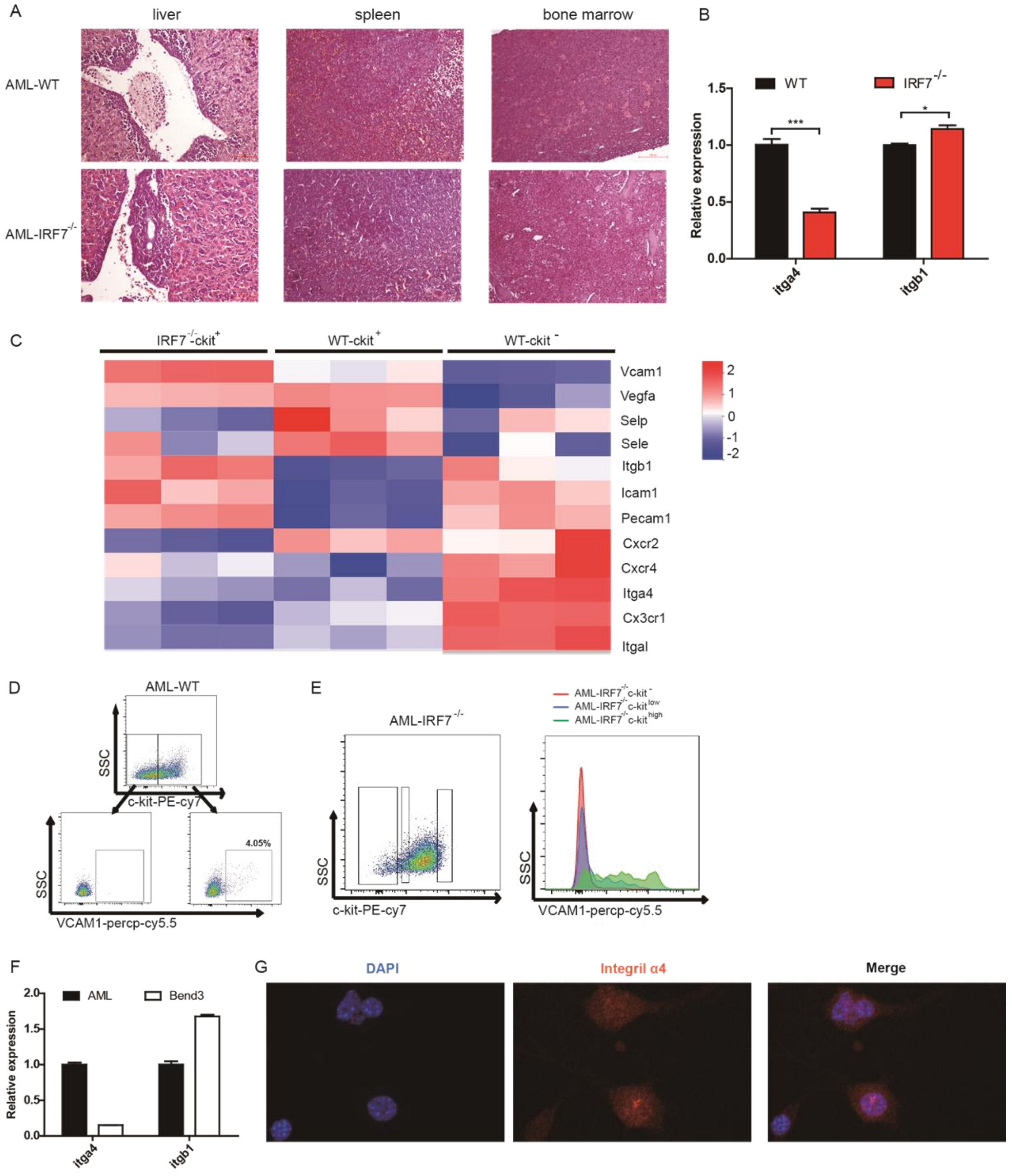

Fig. S9

Supplementary data for enhanced intracerebral invasion of AML-IRF7$^{-/-}$ cells.

(A) Representative HE-stained sections of liver, spleen and bone marrow are shown. (B) The expression of itga4 and itgb1 was analyzed by qRT-PCR. (C) The relative expression of some adhesion molecules and chemokines, which are important for leukocytes to cross the BBB, in AML-WT-c-kit$^-$, AML-WT c-kit$^+$ and AML-IRF7$^{-/-}$ c-kit$^+$ cells is shown. (D) The expression of VCAM1 in AML-WT c-kit$^-$ and AML-WT c-kit$^+$ cells was analyzed by flow cytometry. (E) The median fluorescence intensities (MFI) of VCAM1 in AML-IRF7$^{-/-}$ c-kit$^-$, AML- IRF7$^{-/-}$ c-kit^{low} and

AML- $IRF7^{-/-}$ $c\text{-}kit^{high}$ cells are plotted. (F) The expression of itga4 and itgb1 in Bend.3 cells was analyzed by qRT-PCR. (G) The expression of integrin α4 in Bend.3 cells was analyzed by confocal microscopy. Data are presented as mean ± S.E.M. *$p < 0.05$; *** $p < 0.001$.

Wang H, et al. Figure S10

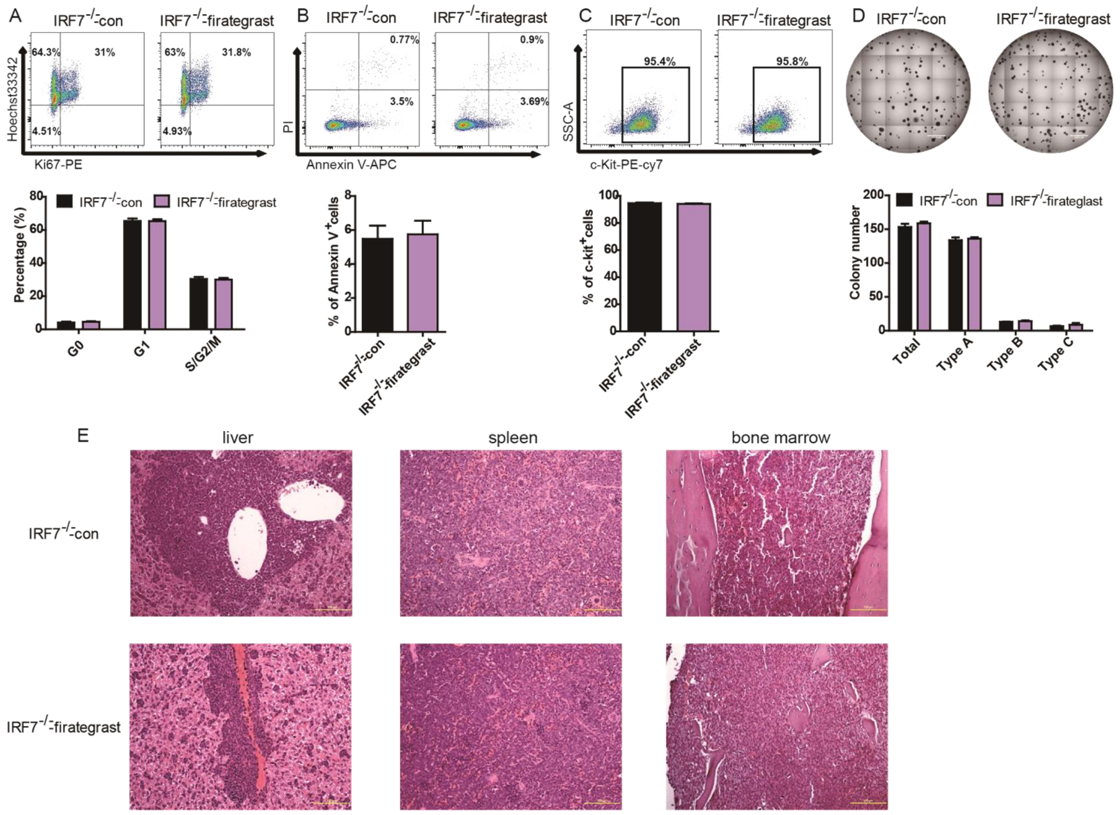

Fig. S10

Supplementary data for the effects of firategrast treatment on AML.

(A-D) Mice were intravenously injected with an equal number of AML-IRF7$^{-/-}$ cells and either received daily firategrast treatment or no treatment. The mice were sacrificed when peripheral blood GFP^{+} cells reached 20%. (A) GFP^{+} leukemia cells were stained with Ki67 and Hoechst 33342. Representative flow cytometry results are shown (upper), and the percentages of G0/G1, S and G2/M phase AML cells are plotted (lower). (B) Representative results of Annexin V and PI staining experiments are shown (upper), and the percentage of apoptotic AML cells is plotted (lower). (C) Representative results of c-kit expression in AML cells are shown (upper), and the percentage of c-kit^{+} AML-IRF7$^{-/-}$ cells is plotted (lower). (D) Colony formation experiments were performed by seeding 500 sorted AML cells per well into 24-well plates. Representative results are shown (upper), and the colony numbers are plotted (lower). (E) Representative HE-stained sections of liver, spleen and bone marrow are shown.
