# Supplementary material for: Loss of IRF7 accelerates acute myeloid leukemia progression and induces VCAM1-VLA-4 mediated intracerebral invasion
Source: Oncogene. 2022 Mar 7;41(16):2303–14. doi: 10.1038/s41388-022-02233-w (PMC9010288; doi:10.1038/s41388-022-02233-w)
Supplement: Supplementary file 2 — supplemental materials [file 41388_2022_2233_MOESM2_ESM.docx]

Supplemental materials

Antibodies and reagents

Antibodies against mouse CD3 (PerCP-Cy5.5), CD19 (APC), Ki67 (PE), c-kit (PE-Cy7), and human Ki67 (PE-Cy7) were purchased from BD (USA). Rabbit IgG (Pol-y4053) and antibodies against mouse VCAM-1 (PerCP-Cy5.5), Annexin V (APC), CD11b (PerCP-Cy5.5), and GR-1 (PE-Cy7) were purchased from Biolegend (USA). Monoclonal antibodies against GAPDH (17#2118) and integrin α4 (8440T) were purchased from Cell Signaling Technology (USA). Polyclonal antibodies against IRF7 (PRS3941) were purchased from Sigma (USA).

RPMI 1640 and DMEM were purchased from Neuronbc (China). M3434 and H3434 were purchased from Stem Cell Technologies (Canada). Fetal bovine serum (FBS), OPTI-MEM, sodium pyruvate, L-glutamine penicillin/streptomycin, nonessential amino acids (NEAAs), and trypsin were purchased from Gibco (USA). The EcoR I and Xho I restriction endonucleases were obtained from New England BioLabs (UK). The SYBR Green PCR Kit was purchased from TaKaRa Biotech (China). IFNα and IFNβ were purchased from Sino Biological (China).

Cell cultural

Human cell lines HEK293T, THP-1, Kasumi-1 and Bend.3 cells were obtained from cell bank of SKLEH (purchased from ATCC, and maintained by cell bank). The cells were tested for mycoplasma contamination monthly using a mycoplasma detection kit (InvivoGen). Cell lines were validated for authentication using the short tandem repeat method (Beijing Microread Corporation Limited). Cells were cultured in RPMI-1640 (THP1 and Kasumi series) or DMEM (HEK293T and Bend.3) supplemented with 10% fetal bovine serum (FBS). All cells were cultured in a humidified incubator at 37 °C in a 5% CO_2_ atmosphere.

Cell cycle analyses

In Ki67 assays, 3×10^6^ cells were fixed and permeabilized following the protocol of the Cytofix/Cytoperm™ Fixation/Permeabilization Solution Kit (BD). Then, the cells were stained with PE-conjugated anti-Ki67 followed by Hoechst 33342 before flow cytometry analysis.

In BrdU incorporation assays, AML mice were intraperitoneally injected with 200 μl BrdU (10 mg/ml). The mice were sacrificed 16 hours later, and 5×10^6^ BM AML cells were sorted and stained with a Pharmingen TM APC BrdU Flow Kit (BD) following the manufacturer’s protocols.

Cell proliferation assay

The 3-(4,5-dimethylthiazol-2-yl)-5-(3-carboxymethoxyphenyl)-2-(4-sulfophenyl)-2H- tetrazolium (MTS) assay was carried out as previously described[2]. Briefly, cells were seeded into 96-well plates at a concentration of 5×10^4^ cells/well. At each time point (including the 0 h control), 20 μl MTS was added to each well, and the cells were incubated for another 2 hours. The absorbance at 490 nm was measured by a microplate reader. Cell proliferation at a time point was assessed as the fold change in absorbance at that time point versus time point 0 h.

Cell apoptosis assay

Cell apoptosis was measured by PI/Annexin V staining as previously described. Briefly, the cells were stained with Annexin V-APC in 1× binding buffer for 15 minutes. PI was added to 1× binding buffer before flow cytometry analysis.

Colony forming assay

Cells were suspended in H3434 (THP1 and Kasumi) or M3434 (mouse AML cells) complete medium (Stem Cell Technologies), plated into 24-well plates (500 cells/500 μl/well), and cultured for 9 days following the manufacturer’s instructions. The colonies of mouse AML cells were classified into three types based on the morphology: the type A colonies had a compact center; the type B colonies had a dense center surrounded by a halo of migrating cells; and type C colonies, which consisted of many diffuse differentiating cells. The colonies were counted and scanned by a high-content analysis system (PerkinElmer, UK).

Western blot

Sample preparation and Western blotting were performed following standard protocols. Briefly, cells were sorted and lysed in RIPA lysis buffer (Cell Signaling Technology, USA) containing phenylmethylsulfonyl fluoride. The BCA Protein Assay Kit (Thermo Scientific, USA) was used to measure the protein concentration. The samples were subjected to SDS-PAGE for electrophoresis and transferred to a polyvinylidene difluoride membrane. Rabbit polyclonal antibodies against IRF7 or mouse monoclonal antibody against GAPDH were used as primary antibodies. Goat-anti-rabbit-HRP or goat-anti-mouse-HRP polyclonal antibodies were used as secondary antibodies. The blot was visualized using chemiDoc (General Electric Company, USA). Data were acquired by gray value analysis (ImageJ).

Nude mice xenograft models

Mice were subcutaneously injected with THP1sc or THP1sh1 cells (1.2×10^7^ in 200 μl PBS) on the right dorsal side. The tumors were measured every 2 days when they were palpable after 10 days. The tumor volume was calculated by the equation: volume = length × width × width/2. The mice were sacrificed on day 17, and the tumors were weighed.

NSG mice xenograft models

NSG mice were irradiated at 2 Gy followed by intravenous injection of THP1sc or THP1sh1 cells (3.5×10^6^ in 300 μl PBS). The survival of mice was recorded. For pathologic analysis of brain tissue, the mice were sacrificed on day 25. Standard hematoxylin-eosin (HE) staining was performed.

Ara-C treatment

To study the effect of Ara-C on leukemia cells, AML-WT or AML-IRF7^-/-^ mice were intraperitoneally administered Ara-C for four days at a dosage of 500 mg/kg/day when PB GFP^+^ cells reached approximately 6%. The PB GFP^+^ cells were monitored every 24 hours. In the survival experiments, AML-WT or AML-IRF7^-/-^ mice were administered Ara-C or PBS on days 13, 14 and 15 after the injection of AML cells. The PB GFP^+^ cells were monitored on days 8, 12, 16 and 20. The survival of mice was recorded.

Type I interferon (Ⅰ-IFN) treatment

Mice were transplanted with 1×10^5^ AML-IRF7^-/-^ cells on day 0. On day 15, the mice were administered Ⅰ-IFN (1×10^4^ U/kg IFNα and 2×10^4^ U/kg IFNβ) by [intraperitoneal](javascript:;) [injection](javascript:;) or did not receive I-IFN. The mice were sacrificed on day 16. The proliferation, apoptosis, colony formation and c-kit expression of AML cells were studied.

Correlation between the expressions of IRF7 and TGIF1

The NCBI GEO datasets (GSE10358, GSE12417 and GSE131207) were downloaded (https://www.ncbi.nlm.nih.gov/geo). GSE10358 contains 304 AML cases. GSE12417 includes 242 cytogenetically normal AML patients and is subdivided into three groups due to different sequencing platforms. The biggest group (137 cases) was used for analysis. GSE131207 contains 76 AML patients, 49 T-ALL patients and 15 healthy donors. The expression of TGIF1 between the IRF7^high^ group (containing the top 30%, 20% or 10% cases) and the IRF7^low^ group (containing the bottom 30%, 20% or 10% cases) was compared.

Microarray analysis

Data for all genes were subjected to gene set enrichment analyses (GSEAs) to investigate the biological functions or pathways. Standard analyses were also performed on the online Majorbio Cloud Platform. Fold change (FC) ≥ 2.0 and p value < 0.05 were used as the cutoff for screening the differentially expressed genes (DEGs). The DEGs between AML-IRF7^-/-^-c-kit^+^ and AML-WT-c-kit^+^ cells are shown in a volcano plot.

Flow cytometry analysis and cell sorting

CantoII ﬂow cytometer and FACS AriaI II (BD Biosciences, USA) were used for FACS analysis and cell sorting, respectively. All experiments were completed following standard protocols. Data analysis was carried out using Diva (BD Biosciences, USA) and Flow JoV_10 (Tree Star, USA) software.

RNA extraction and quantitative reverse-transcription polymerase chain reaction (qRT-PCR)

Total RNA was extracted using TRIzol reagent (Life Technologies, USA) and reverse transcribed using Transcript All-in-one First-Strand cDNA Synthesis SuperMix (TransGen Biotech, China) following the manufacturers’ protocols. qPCR was performed on 0.1-QuantStudio 5 and 0.2-QuantStudio 5 (Thermo Fisher Scientific, USA). The expression level of the target gene was obtained by calculating the RQ value using the ^ΔΔ^Ct method [^ΔΔ^Ct= (Ct_TARGET_−Ct_GAPDH_)_sample_− (Ct_TARGET_−Ct_GAPDH_)_calibrator_].

| Gene | Forward (5’-3’) | Reverse (3’-5’) |
| --- | --- | --- |
| mGAPDH | TGAAGGTCGGTGTGAACGGATT | CTCGCTCCTGGAAGATGGTGAT |
| mIRF7 | GTCACCACACTACACCATCTACCT | TAGACAAGCACAAGCCGAGACT |
| mTGIF1 | GCAAGAGAAGGAGGAGAGGCAAT | GGCGTTGATGAACCAGTTACAGAC |
| mRAB34 | CTGATGCCCTCAAGGAGAACGA | GCTGCCACACGGAAGAAGAATT |
| mCREBL2 | CACTCCGAGAGGAACTGGAAATGTA | GTTGGTGTCAGCGTCTGTCTTC |
| mE2F2 | GCGAGTCGGAGGATGGAGTC | CTTGTTGGCATTATCTTCGGTCAGG |
| mTHBS1 | AAGACTGTGTTGGCGATGTGAC | ACCGATGTTCTCCGTTGTGATTG |
| mDIXDC1 | AGAACAGAACAGATGAGCCAGACT | GTCCTGATTCTCCTCCACACTCT |
| mVCAM1 | GTGCTGCTATTGGCTGTGACTC | GACCTCCACCTGGGTTCTCTTT |
| mVEGFa | CACGACAGAAGGAGAGCAGAAGT | GCTGGCTTTGGTGAGGTTTGAT |
| mICAM1 | CGCAGAGGACCTTAACAGTCTACAA | TCCAGCCGAGGACCATACAG |
| mPECAM1 | TCCTTCACCATCAACAGCATCCATA | ACCATCGCATCGTCCTTATAGAACA |
| mItga4 | CGACTTGAGAGGTGCTGTCTACAT | ACCAACGGCTACATCAACATATCCA |
| mItgb1 | AGGACATTGATGACTGCTGGTTCT | CACAGTTGTCACGGCACTCTTG |
| hGAPDH | GAAGGTGAAGGTCGGAGTC | GAAGATGGTGATGGGATTTC |
| hIRF7 | GAAGAGCCTGGTCCTGGTGAA | GGAAGCACTCGATGTCGTCATAGA |
| hTGIF1 | GAAGAGCCTGGTCCTGGTGAA | GGAAGCACTCGATGTCGTCATAGA |
